# Non-Invasive Tools for the Diagnosis of Potentially Life-Threatening Gynaecological Emergencies: A Systematic Review

**DOI:** 10.1371/journal.pone.0114189

**Published:** 2015-02-27

**Authors:** Viola Polena, Cyrille Huchon, Catalina Varas Ramos, Roman Rouzier, Alexandre Dumont, Arnaud Fauconnier

**Affiliations:** 1 EA 7285 Research Unit “Risk and Safety in Clinical Medicine for Women and Perinatal Health”, Versailles-Saint-Quentin University (UVSQ), 78180, Montigny-le-Bretonneux, France; 2 Department of Gynaecology and Obstetrics, Intercommunal Hospital Centre of Poissy-Saint-Germain-en-Laye, 78103, Poissy, France; 3 Department of Surgery, Institut Curie, 35 rue Dailly, 92210, Saint-Cloud, France; 4 Institut de Recherche pour le Développement, UMR 216, Université Paris Descartes, Sorbonne Paris Cité, Paris, France; Oslo University Hospital, Ullevål, NORWAY

## Abstract

**Objective:**

To identify non-invasive tools for diagnosis of the major potentially life-threatening gynaecological emergencies (G-PLEs) reported in previous studies, and to assess their diagnostic accuracy.

**Methods:**

MEDLINE; EMBASE; Cochrane Central Register of Controlled Trials (CENTRAL; The Cochrane Library) were searched to identify all eligible studies published in English or French between January 1990 and December 2012. Studies were considered eligible if they were primary diagnostic studies of any designs, with a gold standard and with sufficient information for construction of a 2 × 2 contingency table, concerning at least one of the following G-PLEs: complicated ectopic pregnancy, complicated pelvic inflammatory disease, adnexal torsion and haemoperitoneum of any gynaecological origin. Extraction of data and assessment of study quality were conducted by two independent reviewers. We set the thresholds for the diagnostic value of signs retrieved at Sensibility ≥ 95% and LR—≤ 0.25, or Specificity ≥ 90% and LR+ ≥ 4.

**Results:**

We identified 8288 reports of diagnostic studies for the selected G-PLEs, 45 of which met the inclusion criteria. The methodological quality of the included studies was generally low. The most common diagnostic tools evaluated were transvaginal ultrasound (20/45), followed by medical history (18/45), clinical examination (15/45) and laboratory tests (14/45). Standardised questioning about symptoms, systolic blood pressure<110 mmHg, shock index>0.85, identification of a mass by abdominal palpation or vaginal examination, haemoglobin concentration <10 g/dl and six ultrasound and Doppler signs presented high performances for the diagnosis of G-PLEs. Transvaginal ultrasound was the diagnostic tool with the best individual performance for the diagnosis of all G-PLEs.

**Conclusion:**

This systematic review suggests that blood pressure measurement, haemoglobin tests and transvaginal ultrasound are cornerstone examinations for the diagnosis of G-PLEs that should be available in all gynaecological emergency care services. Standardised questioning about symptoms could be used for triage of patients.

## Introduction

The main objective of gynaecological emergency care services is to identify patients at high risk, whose conditions may deteriorate rapidly [1–3] and pose a potential threat to their life or fertility [4–12]. Healthcare services must have efficient screening processes for these patients, and any deficiency in this respect may have a major impact, by delaying therapeutic management, particularly for early pregnancy complications [3–6, 8, 12–14].

Complicated ectopic pregnancy (C-EP; laparoscopic or laparotomy visualization of a tubal wall rupture or active bleeding), adnexal torsion (AT), complicated pelvic inflammatory disease (C-PID; tubo-ovarian abscess and pyosalpinx) and haemoperitoneum (HmPT) of any gynaecological origin are typical potentially life-threatening gynaecological emergencies (G-PLE) [3–5, 9–23]. The late diagnosis and inappropriate management of these acute conditions may lead to severe morbidity and death [5, 12].

Conversely, pelvic pain and vaginal bleeding are the most frequent symptoms reported by women during emergency visits. Accurate diagnosis is difficult for patients with acute pelvic pain, making it difficult to take decisions concerning the management of these patients. Unnecessary surgical explorations increase the risk of morbidity.

Various non-invasive methods, such as clinical examination, pelvic imaging and laboratory tests, have been proposed and are routinely used in clinical practice for the diagnosis for G-PLEs [6–12]. Disparities exist in the provision of diagnostic services, due to differences in the care models applied. The diagnostic value of the tools used has yet to be systematically evaluated and summarised. The diagnostic methods and resources used in emergency departments require further exploration, for the development of comprehensive diagnostic strategies.

**The purpose of this systematic review** was to identify non-invasive tools for the diagnosis of G-PLEs reported in previous studies and to assess their diagnostic accuracy, to promote an evidence-based strategy for improving patient outcomes.

## Methods

### Sources and study selection

**Inclusion criteria**. Studies were considered eligible if they were diagnostic studies for G-PLE of any design, with a gold standard. G-PLEs are defined here as acute gynaecological conditions responsible for pelvic pain that might spontaneously become life-threatening or cause sequelae (organ failure or removal) in the absence of prompt diagnosis and treatment. We focused on four major G-PLEs: complicated (ruptured) ectopic pregnancy (C-EP), complicated pelvic inflammatory disease (C-PID: tubo-ovarian abscess and pyosalpinx), adnexal torsion (AT) and haemoperitoneum (HmPT) of any gynaecological origin.

The following inclusion criteria were used to select articles for this review: (1) original studies evaluating at least one non-invasive diagnostic tool (history-taking, physical examination, ultrasound, MRI, CT scan, biological tests) in women of reproductive age with suspected G-PLEs, (2) studies reporting sufficient information for construction of a 2 × 2 contingency table, (3) visualisation of the G-PLEs (adnexal torsion, ruptured ectopic pregnancy, tubo-ovarian abscess, pyosalpinx and haemoperitoneum) at surgery (laparoscopy or laparotomy) as the gold standard, with or without histological confirmation, and appropriate clinical follow-up to check that G-PLEs were not missed.

The exclusion criteria were as follows: studies including fewer than 10 patients; study report not in English or French; short communication, abstract for which the full-text article was unavailable, or systematic reviews; studies in premenarchal patients; studies not providing sufficient information to derive 2x2 contingency tables for test results and the final diagnosis of at least one G-PLE; secondary analysis of an included study. Studies with a primary focus on gynaecological emergency but with no information about G-PLE status (e.g. studies limited to ectopic pregnancies without specifying rupture or haemoperitoneum status; studies limited to pelvic inflammatory disease without specifying whether there were complications in the form of abscess or pyosalpinx) were also excluded.

**Search methods**. We searched MEDLINE, EMBASE and the Cochrane Central Register of Controlled Trials (CENTRAL; The Cochrane Library) for diagnostic studies, published in English or French between January 1990 and December 2012, with the following search string: (“gynaecological emergency” [MesSH] AND “diagnosis” [MeSH]) OR specific terms concerning different G-PLEs). The full strategies used to search MEDLINE, EMBASE, and CENTRAL are outlined in Appendices 1, 2, and 3 in S1 File.

All the retrieved studies were identified in PubMed, in which we used the “related articles” feature to identify additional published articles. We checked the Science Citation Index, to identify articles citing the studies selected for our review. Two of us (VP, CVR) read the reference lists of articles relevant to our topic, to identify additional studies.

**Study selection**. One of us (VP) performed the searches, and all the titles and abstracts retrieved were downloaded to the reference management database. Duplicates were removed and the same author (VP) examined the remaining references and read the titles and abstracts to determine whether they met the inclusion and exclusion criteria. One of us (VP) then read the full text of eligible articles to determine whether they met our inclusion and exclusion criteria.

### Data extraction and management

Two of us (VP, CVR), working independently, used a standardised data collection form to extract data from each selected study. The data collection form was first tested on three studies and was modified by an iterative process involving discussion between the authors. The following data were extracted from each study: study identification, study design and general characteristics, study population, inclusion and exclusion criteria, settings, summary details of the index test (i.e. sign) used to detect G-PLE, reference standards, flow of patients, data for the construction of 2 x 2 contingency tables for G-PLE outcomes for the various diagnostic tools evaluated and QUADAS judgments.

We assessed the methodological quality of all the included studies. We classified the risk of bias and applicability as “low”, “unclear”, or “high” with the QUADAS 2 tool (Quality Assessment of Diagnostic Accuracy Studies) [24], and disagreements between reviewers were resolved by discussion to reach a consensus.

Review Manager version 5.1 software was used for data entry. The results are reported as both a methodological quality graph and a methodological quality summary.

### Statistical analysis

Sensitivity (Se), specificity (Sp), positive likelihood ratio (LR+) and negative likelihood ratio (LR-) were calculated for all symptoms or signs analysed. The values obtained were grouped together by diagnostic tool (e.g. transvaginal ultrasound, bimanual examination) to determine the overall diagnostic accuracy of the test. Continuous variables are expressed as medians (first quartile, third quartile) and ranges (minimum and maximum values) and categorical variables are expressed as frequencies and percentages.

Only diagnostic tests found to have a specificity of at least 90%, with a sensitivity high enough to provide an overall positive likelihood ratio of at least 4.0 or with a sensitivity remaining at or over 95% while maximising specificity to give a negative likelihood ratio of no more than 0.25 were considered to be clinically effective [25, 26]. Microsoft Excel version 14.4.3 and COCHRANE Review Manager 5.1 were used for statistical analysis.

A protocol of the systematic review was not published. Therefore, the protocol can be obtained by contacting the authors.

## Results

### Study identification

**Fig 1:**
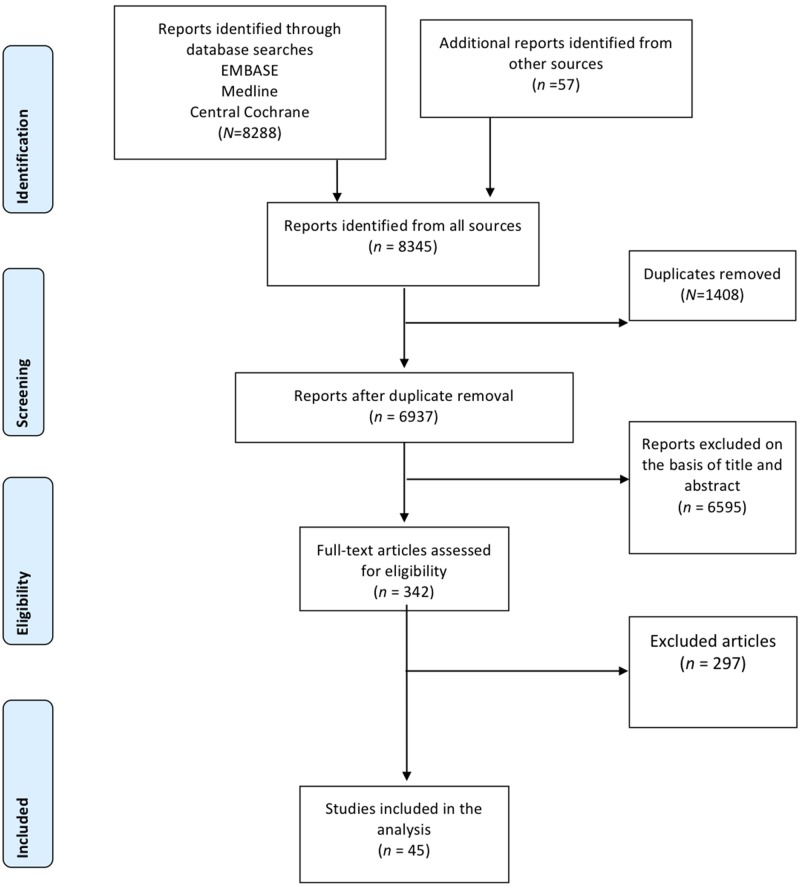
Flow-chart for study selection.

Fig. 1 shows the study selection flow chart. We identified 8288 diagnostic studies for G-PLEs, 342 of which were selected on the basis of their titles and abstracts. The full articles for these 342 relevant studies were reviewed and the selection criteria applied. This process yielded a final list of 45 articles suitable for systematic review. The references of the included studies are provided in Appendix 4 in S1 File. Appendices 5, 6, 7, 8 in S1 File contain the study selection flow charts for each G-PLE.

### Methodological quality of the included studies

**Fig 2:**
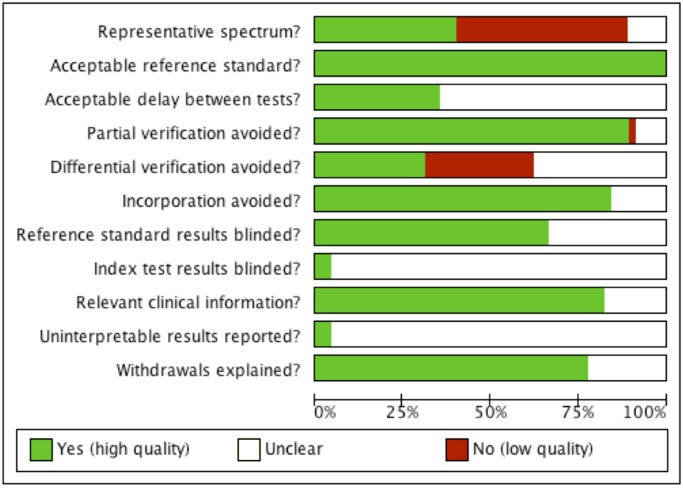
Methodological quality graph. Our judgements for each methodological quality item are presented as percentages for all included studies.

**Fig 3:**
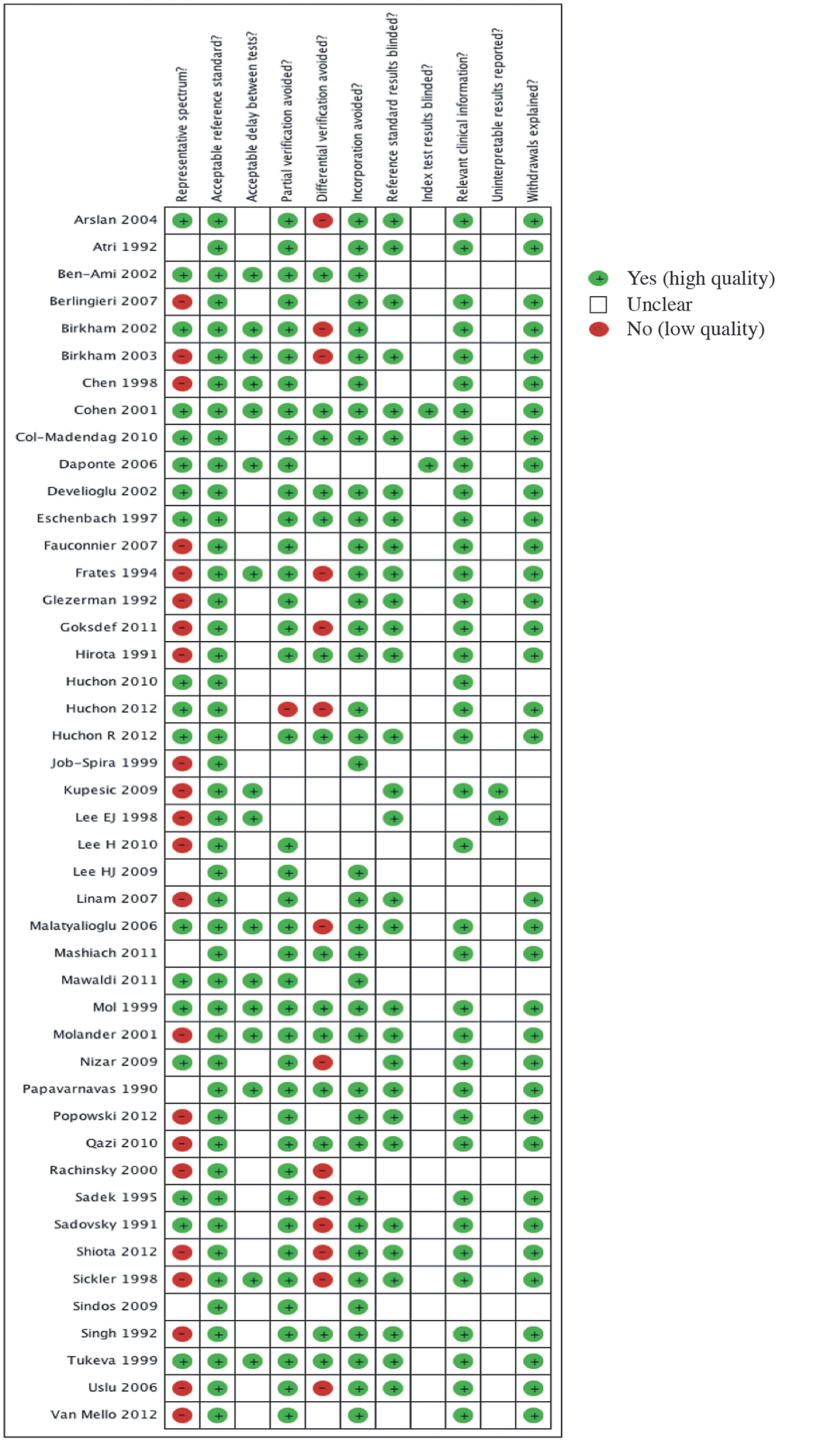
Methodological quality graph. Our judgements for each methodological quality item for all individual studies.

Our judgements for each methodological quality item are presented in methodological quality graphs (Fig. 2: summary; and Fig. 3: individual studies). Methodological quality estimates were based on the study reports. Most of the studies did not include a representative spectrum of patients or the reporting was unclear (Figs. 2 and 3). Almost half the studies (22/45) presented a high risk of spectrum bias. In these studies, patients were mostly included on the basis of surgical results. Most of the studies (43/45) did not specify whether the index test results were blinded. It was also not possible to ascertain from the included studies whether the results of the gold standard test were blind.

### Description of studies

Table 1 reports the characteristics of the studies included in our review. Various study designs were used. Most of the studies were retrospective (24, 53%) or their nature was unclear (3, 7%). Only 18 (40%) were prospective. Less than 16% (7/45) of the studies involved more than one study centre. Most of the studies were conducted in gynaecological emergency units (33/45).

**Table 1:** Characteristics of the 45 studies included in our review.

	G-PLE^1^ (*N* = 45)	AT^2^ (*N* = 14; 31%)	HmPT^3^ (*N* = 5; 11%)	C-EP^4^ (*N* = 20; 45%)	C-PID^5^ (N = 6; 13%)
**Type of study *N* (%)**					
Retrospective	24 (53.3)	8 (57.1)	5 (100.0)	11 (55.0)	1 (16,7)
Prospective	18 (40.0)	5 (35.7)	0	9 (45.0)	3 (50,0)
Unclear	3 (6.7)	1 (7.2)	0	0	2 (33,3)
**Number of centres**					
Single centre	35 (77.7)	11 (78.6)	3 (60.0)	15 (75.0)	6 (100,0)
Multicentre	7 (15.6)	1 (7.1)	1 (20.0)	5 (25.0)	0
Unclear	3 (6.7)	2 (14.3)	1 (20.0)	0	0
**Type of centre**					
Gynaecological	33 (73.4)	10 (71.4)	4 (80.0)	15 (75.0)	4 (66,7)
Other	12 (26.6)	4 (28.6)	1 (20.0)	5 (25.0)	2 (33,3)
**Total number of patients**	6885	1707	867	4682	209
(Med: Q1; Q3)	(98: 47; 215)	(64: 53; 131)	(185: 89; 215)	(146: 87; 254)	(26: 21; 38)
**Disease**	NA	444	569	1292	108
Disease-free	NA	1263	298	3390	101
**Patient age**	30 (7; 83)	32 (7; 83)	30.2 (15; 44)	29.5 (15; 46)	34 (15; 53)
**Types of non-invasive tool**					
**Medical history**	18 (40.0)	3 (21.4)	2 (40.0)	12 (60.0)	1 (16,6)
(Number of studies; %)					
**Clinical examination**	15 (33.0)	3 (21.4)	3 (60.0)	7 (35.0)	2 (33,3)
**Imaging**	23 (51.0)	9 (64.3)	4 (80.0)	6 (30.0)	4 (66,7)
TVUS^6^	20 (44.0)	7 (50.0)	4 (80.0)	6 (30.0)	4 (66,7)
TAUS^7^	2 (4.4)	1 (7.1)	1 (20.0)	0	0
CT scan	1 (2.2)	1 (7.1)	0	0	0
MRI	1 (2.2)	0	0	0	1 (16,6)
Scintigraphy	2 (4.4)	0	0	0	2 (33,3)
**Laboratory**	14 (31.0)	3 (21.4)	2 (40.0)	8 (40.0)	1 (16,6)

(Data presented as number of studies; %)

^**1.**^ **Potentially life-threatening gynaecological emergency;**

^**2.**^ **Adnexal torsion;**

^**3.**^ **Haemoperitoneum;**

^**4.**^ **Complicated ectopic pregnancy;**

^**5.**^ **Complicated pelvic inflammatory disease;**

^**6.**^ **TVUS = transvaginal ultrasound;**

^**7.**^ **TAUS = transabdominal ultrasound**

The inclusion criteria differed between studies: acute pelvic pain (30/45) and/or vaginal bleeding (8/45) were the most frequent indications. Other indications included ovarian cyst complications, early pregnancy complications.

Reports focused on AT in 14 studies (31%), C-EP in 20 studies (45%), haemoperitoneum in 5 studies (11%), and C-PID in 6 studies (13%).

In total, 6885 women were included in the 45 studies: 1707 women were included in AT studies (444 AT), 867 in haemoperitoneum studies (569 HmPT), 4682 in ruptured ectopic pregnancy studies (1292 C-EP), and 209 in C-PID studies (108 C-PID). The median age of the women included in these studies was 30 years (range: 7; 83 years).

Medical history, clinical examination, imaging and laboratory tests were reported as diagnostic tests in these studies (Table 1). The most common diagnostic tool evaluated was transvaginal ultrasound (20/45), followed by medical history (18/45). Clinical examination (vital signs, abdominal palpation, bimanual examination) was evaluated in 15 studies (33%) and laboratory tests (blood formula, B-hCG, CRP concentration etc.) were evaluated in 14 studies (31%).

Most of the reports described the tools used to diagnose G-PLE. However, none provided information about the number of clinicians performing the diagnostic tests or their experience, and none described the specific techniques used, except for ultrasound. In most of the studies involving ultrasound, the qualification of the clinicians performing the imaging procedure was not specified (12/22); ultrasound scans were carried out by a gynaecologist (resident or consultant) in six studies, and by a radiologists in another four studies.

**Signs**. Diverse signs were described during the performance of the clinical, ultrasonographic and biological tests and the interpretation of their results (Appendices 9, 10, 11, 12 in S1 File). Most of the studies reported the use of transvaginal ultrasound, transabdominal ultrasound was used in two studies.

Table 2 reports the estimated accuracy of signs/symptoms with diagnostic performances considered to be of clinical utility. Reports of syncope, unilateral abdominal pain, ovarian stimulation and pain during movement during history-taking were considered highly suggestive of G-PLEs. Systolic blood pressure <110 mmHg, shock index >0.85 (the ratio of heart rate to systolic blood pressure), identification of a mass by abdominal palpation or vaginal examination, haemoglobin concentration <10 g/dl, and six ultrasound and Doppler signs were considered to perform well for the diagnosis of G-PLEs (Table 2). The following ultrasound signs were of high diagnostic value: tube wall thickness > 5 mm, cogwheel sign for the diagnosis of C-PID; abnormal Doppler scan for the diagnosis of C-PID and AT; echogenic fluid, fluid in the Morison pouch, trilaminar endometrial pattern and foetal cardiac activity for HmPT and C-EP.

**Table 2:** Non-invasive tools: Accuracy of signs.

Findings	Condition	Study	Type of study	No. of patients (D+/D-)^1^	Se	SP	LR+	LR-
**History-taking**								
Ovarian stimulation	**AT** ^**2**^	Huchon 2010	R^6^	142 (32/110)	0.56	0.96	4.30	0.88
Syncope	**C-EP** ^**3**^	Huchon 2012	P^7^	141 (30/111)	0.27	0.97	7.99	0.76
Pain during movement	**C-EP**	Huchon 2012	P	141 (30/111)	0.96	0.33	1.43	0.11
Unilateral abdominal or lumbar pain	**AT**	Huchon 2012	P	482 (31/451)	0.97	0.50	1.90	0.07
**Physical examination**								
Palpation-adnexal mass	**C-PID** ^**4**^	Eschenbach 1997	P	66 (8/58)	0.75	0.95	14.5	0.26
Systolic blood pressure < 100 mmHg	**HmPT** ^**5**^	Fauconnier (2007)	R	89 (48/41)	0.21	0.95	4.27	0.83
Systolic blood pressure < 100 mmHg	**C-EP**	Birkham 2002	P	280 (24/256)	0.17	0.98	8.53	0.85
Shock index > 0.85	**C-EP**	Birkham 2002	P	280 (24/256)	0.42	0.97	13.33	0.60
Bimanual examination—irregular mass	**C-EP**	Qazi 2010	R	50 (44/6)	0.89	0.83	5.32	0.14
	**HmPT**	Fauconnier (2007)	R	89 (48/41)	0.17	0.98	6.83	0.85
	**HmPT**	Popowski (2012)	R	215 (48/167)	0.57	0.93	7.68	0.46
	**HmPT**	Sickler (1998)	R	185 (122/63)	1.00	0.95	21.00	0.00
	**C-EP**	Sadek (1995)	P	525 (5/520)	1.00	0.91	10.61	0.00
**Abdominal ultrasound**								
Fluid in Morison pouch	**HmPT (>500 ml)**	Popowski (2012)	R	215 (48/167)	0.52	0.92	6.69	0.52
	**HmPT (>100 ml)**	Popowski (2012)	R	215 (108/107)	0.29	0.98	15.36	0.73
**Transvaginal ultrasound**								
Tube wall thickness > 5 mm	**C-PID**	Molander (2001)	R	34 (14/20)	1.00	0.90	10.00	0.00
Cogwheel sign	**C-PID**	Molander (2001)	R	34 (14/20)	0.79	0.95	15.71	0.23
Echogenic fluid	**HmPT**	Chen (1998)	R	46 (37/9)	0.95	1.00		0.05
	**HmPT**	Fauconnier (2007)	R	89 (48/41)	0.17	0.98	6.83	0.85
	**HmPT**	Popowski (2012)	R	215 (48/167)	0.57	0.93	7.68	0.46
	**HmPT**	Sickler (1998)	R	185 (122/63)	1.00	0.95	21.00	0.00
	**C-EP**	Sadek (1995)	P	525 (5/520)	1.00	0.91	10.61	0.00
Endometrial pattern: Trilaminar	**C-EP**	Col-Madendag (2010)	R	99 (30/69)	0.60	0.96	13.80	0.42
Foetal cardiac activity	**C-EP**	Mol (1999)	P	252 (65/187)	0.23	0.98	14.38	0.78
**Doppler**								
Abnormal Doppler	**C-PID**	Molander (2001)	R	34 (14/20)	1.00	0.90	10.00	0.00
	**AT**	Ben-Ami 2002	P	65 (15/50)	1.00	0.98	50.00	0.00
	**AT**	Kupesic 2010	R	36 (24/12)	0.88	1.00		0.13
	**AT**	Lee E 1998	Unclear	28 (12/16)	1.00	0.94	16.00	0.00
	**AT**	Nizar 2009	P	193 (29/164)	0.76	0.99	62.21	0.24
**Biological tests**								
Haemoglobin concentration <10 g/dl	**HmPT**	Fauconnier 2007	R	89 (48/41)	0.29	0.98	11.96	0.73
	**HmPT**	Popowski (2012)	R	215 (48/167)	0.31	0.95	6.52	0.72
	**C-EP**	Mol 1999	P	252 (65/187)	0.23	0.98	14.38	0.78
**Diagnostic models**								
Composite score>60	**AT**	Huchon 2010	P	35 (6/29)	0.50	0.97	14.50	0.51
Standardised questionnaire	**C-EP**	Huchon 2012	P	141 (30/111)	0.27	0.97	9.90	0.75
Torsion score>6	**AT**	Huchon 2012	P	496 (31/465)	0.97	0.70	3.26	0.05
Torsion score = 10	**AT**	Huchon 2012	P	496 (31/465)	0.39	0.98	16.40	0.63

^1.^ Disease +/ Disease-;

^2.^ Adnexal Torsion;

^3.^ Complicated ectopic pregnancy;

^4.^ Complicated pelvic inflammatory disease;

^5.^ Haemoperitoneum;

^6.^ Retrospective;

^7.^ Prospective

Abnormal Doppler findings were strongly suggestive of adnexal torsion, with a high sensitivity (range: 76%; 100%) and specificity (range: 94%; 100%), while free pelvic fluid was highly suggestive of a ruptured ectopic pregnancy or haemoperitoneum, with a specificity of 91% to 100%, depending on the study considered. Vital signs, systolic blood pressure < 100 mmHg and shock index > 0.85 (specificity range: 95%; 98%), and a Hb concentration < 10 g/dl were highly specific for the prediction of ruptured ectopic pregnancy and haemoperitoneum.

In overall analyses of the individual diagnostic tools, transvaginal ultrasound was found to be the diagnostic tool with the highest performance for the diagnosis of G-PLEs. Transvaginal ultrasound has both high sensitivity and high specificity. By contrast, clinical and biological tests had a low sensitivity but a high specificity.

## Discussion

Our assessment indicated that the assessment of vital signs, clinical examination, ultrasound and haemoglobin tests are important diagnostic tools for first-line examination in female patients of reproductive age with acute pelvic pain or bleeding, to ensure the accurate detection of G-PLEs. Transvaginal ultrasound was the individual diagnostic tool with the best performance for the diagnosis of all G-PLEs. Standardised questioning may be useful for triage, but all the studies dealing with this approach were published by the same team.

### Strengths and limitations of the study

This is, to our knowledge, the first systematic review of the performance characteristics of non-invasive diagnostic tools for potentially life-threatening gynaecological emergencies. Our study is original in that we considered the identification, not of a single disease, but of the most representative high-risk conditions encountered in gynaecological emergency departments and requiring prompt management, to prevent severe complications with a long-term impact on the health and well-being of women. A global view of high-risk situations is essential, to determine the best approaches for diagnosis and to identify the facilities required by gynaecological emergency services. However, a meta-analysis was not possible, due to the considerable heterogeneity of the studies in terms of outcomes, diagnostic signs and diseases. We currently have access only to studies considering different types of G-PLEs separately. It would therefore be inappropriate to pool the performances of tests for different components of a composite outcome. A meta-analysis would, however, be possible if there were primary studies evaluating diagnostic performances for the composite outcome (G-PLEs).

One of the limitations of this study is the limited search period for published studies, given that ultrasound scans were not introduced until the 1990s. This short search period may have led to a bias against studies evaluating clinical examination. Indeed, the most common diagnostic tool evaluated was transvaginal ultrasound (44% of the studies), followed by medical history (40% of the studies). Clinical examination (vital signs, abdominal palpation, bimanual examination) was evaluated only in 15 studies (33%).

Another limitation of this review is the poor methodological quality of the studies included. We identified a number of factors potentially causing bias in the studies included: no representative spectrum, no blinding of the index and reference tests and differential verification. These factors may have generated two different types of bias that must be taken into account when interpreting our results: the spectrum effect and verification bias. Almost half the studies (22/45) presented a high risk of spectrum effect. The performance of a medical diagnostic test may vary as a function of the severity and clinical presentation of the disease within a subgroup of patients. In most of the studies included, the patients were selected essentially on the basis of surgical results. Studies involving the recruitment of participants with a known disease status (Molander 2001, Kupesic 2010, Chen 1998) may report different accuracy results from studies recruiting a cohort of patients without selection on the basis of disease status (Mol 1999, Nizar 2009, Huchon 2012) and representative of the clinical population in which the test is used. This situation might result in the estimated accuracy not being applicable for the clinical condition concerned in some cases [27].

Verification bias is a common feature of diagnostic research, with results for a diagnostic gold standard being available primarily for patients also testing positive in the test studied [28]. All the studies included in this analysis applied a consistent reference standard but, in most studies, there was no blinding of index test results. However, it remains unclear whether the results of the gold standard test were independent of the index test. This probably resulted in a form of verification bias [29]. Due to the nature of the diagnostic tests for G-PLEs and the invasive nature of the gold standard (surgery) for these conditions, differential verification occurs in the general population of women presenting with gynaecological emergencies, as most women with diagnostic test results inconsistent with a high-risk situation will be sent home or have their screening tests verified later, rather than immediately by surgery. This can artificially increase the sensitivity and decrease the specificity values obtained for the diagnostic tool [30].

### Generalisation of the results of the study

One of the key limitations of this review in terms of the generalisation of results, is the lack of information about the qualifications and experience of the clinicians performing the diagnostic tests. For transvaginal ultrasound, for example, the examination is likely to be more accurate if performed by a physician with board certification in gynaecological ultrasound examination. However, the availability of board-certified ultrasound operators is limited in gynaecological emergency departments, and transvaginal ultrasound examinations are routinely performed by diverse first-line physicians without specific expertise in this technique [31,32].

Most of the studies were conducted in gynaecological departments or gynaecological emergency departments. The results of these studies are therefore almost certainly applicable to other departments of these kinds.

The value of medical history [33–37], clinical examination [37–46], ultrasound [47–73], and laboratory tests [70–73]_,_ for the diagnosis of acute gynaecological conditions has been widely reported in many studies. However, there have been few systematic reviews and meta-analyses, and most of the available studies have focused on transvaginal ultrasound examinations for the diagnosis of ectopic pregnancy. Two previous systematic reviews [47, 48] investigated the value of ultrasound scans performed by emergency doctors for the identification of ectopic pregnancy in patients with first-trimester pelvic pain or bleeding. The accuracy of these examinations was good, but the authors of these studies were unable to generate summary statistics because of heterogeneity. A third study [49] also investigated the value of transvaginal ultrasound scans performed by an emergency physician for the detection of ectopic pregnancy. However, by contrast to the other two studies, this study used transvaginal ultrasound as a screening test to rule out ectopic pregnancy.

Crochet *et al*. [50] carried out a systematic review assessing the accuracy and precision of patient history, clinical examination, available laboratory test values, and ultrasonography for the diagnosis of ectopic pregnancy in women with abdominal pain or vaginal bleeding early in pregnancy. They found that all components of patient history had a positive likelihood ratio below 1.5. The most important finding of this study was that a lack of adnexal abnormalities on transvaginal ultrasound scans (LR- 0.12; 95% CI, 0.03–0.55; *n* = 6885) was associated with a lower likelihood of ectopic pregnancy.

### Implications for practice

All G-PLEs can be treated effectively with basic healthcare resources [4, 5, 74–77], but late diagnosis, potentially leading to progression to severe morbidity or death, is a matter of concern. The early diagnosis of ectopic pregnancy is known to decrease the probability of tubal rupture [5, 12, 27, 78]. Acute pelvic pain, the chief complaint evaluated in gynaecological emergency departments, is the most common symptom of G-PLEs. Many conditions cause acute abdominal pain and no single clinical finding or test is both specific and sensitive.

Vital signs (systolic blood pressure < 100 mmHg and shock index > 0.85) are helpful for the identification of high-risk patients, but they are not sufficient to exclude potentially life-threatening gynaecological conditions.

The use of diagnostic ultrasonography for gynaecological emergency diagnosis has greatly increased over the last 20 years. However, survey data suggest that, in many healthcare settings, and particularly in the United Kingdom, emergency access to ultrasonography is still unreliable. This problem concerns women who are not pregnant in particular, because women with early pregnancy problems are seen exclusively in stand-alone units with immediate access to ultrasound [49]. Women with acute pelvic pain should be examined by ultrasound whether or not they pregnant. It has been shown that the availability of transvaginal ultrasound at the initial assessment of both pregnant and non-pregnant women decreases time to patient management, unnecessary admissions and outpatient follow-up examinations, and modifies treatment decisions [79, 80]. We have also shown, in a retrospective study, that having transvaginal ultrasound facilities available round the clock for first-line investigations in addition to physical examination might be an effective strategy for ruling out G-PLEs in gynaecological emergency departments, by decreasing the risk of diagnostic errors [32].

Another question concerns the abilities of non-radiologists to perform ultrasound examinations. In several studies dealing with gynaecological emergencies, non-radiologists performed the ultrasound examination. It remains unclear which factors have the greatest impact on the accuracy of ultrasound examinations. Teaching models and standardised methods are being developed and will probably improve the competence of residents in the performance of emergency ultrasound examinations [81].

This systematic review suggests that ultrasound facilities should be available round the clock for gynaecological emergency department patients. The potential impact of standardised programs with quality control on diagnostic accuracy and patient outcomes should be investigated further.

Medical history and symptoms are not very reliable for the diagnosis of G-PLEs. However, if combined in predictive models, they might improve the selection of patients with suspected G-PLEs for diagnostic surgery and could be used as a triage strategy. The originality of the triage models developed by our research team [81–84] lies in the exclusive use of standardised self-assessed questionnaires without the need for intervention from healthcare professionals. The use of these models could improve patient care in emergency departments, by decreasing delays in patient management. These models require validation in large prospective studies.

### Unanswered questions and future research

When developing screening policies or a clinical diagnostic strategy, clinicians and policy makers may need to make decisions about the types of tests offered to patients. There is therefore a need to define the optimal modes of diagnosis for G-PLEs. The main goal is the effective prevention of severe morbidity and mortality.

It would be of interest to develop and evaluate decision tools combining different clinical findings rather than based on a single test, to improve the management of G-PLEs. However, there is currently no consensus concerning the appropriateness of such tools for clinical use.

The definition of a diagnostic model for G-PLEs could potentially decrease morbidity and mortality and reduce the costs associated with repeated emergency department visits, hospitalisation, radical surgery, and subsequent infertility problems.

## Conclusions

The results of this systematic review suggest that blood pressure measurement, ultrasound examinations, including transvaginal examinations, and haemoglobin tests should be made available in gynaecological emergency care services, to facilitate the detection of G-PLEs. Ultrasound scans are highly accurate for the detection of potentially life-threatening gynaecological emergencies. The assessment of medical history by standardised questioning may be useful for triage purposes, facilitating the selection of patients requiring more rapid access to these diagnostic tools. Further studies are required to assess the implementation of these diagnostic tools and their impact on health outcomes, when used alone or in combination.

## Supporting Information

S1 File Appendices 1–12 and PRISMA Checklist. (DOC)
