# Supplementary material for: Non-Invasive Tools for the Diagnosis of Potentially Life-Threatening Gynaecological Emergencies: A Systematic Review
Source: PLoS One. 2015 Feb 27;10(2):e0114189. doi: 10.1371/journal.pone.0114189 (PMC4344336; doi:10.1371/journal.pone.0114189)
Supplement: S1 File — (DOC) [file pone.0114189.s001.doc]

File SI. Appendices 1-12 and PRISMA Checklist

**Appendix 1**

**MEDLINE search strategy**

Medline 1990 December 2012

## Adnexal torsion

(Adnexal[All Fields] AND torsion[All Fields] AND ("diagnosis"[Subheading] OR "diagnosis"[All Fields] OR "diagnosis"[MeSH Terms])) AND (("1990/01/01"[PDAT] : "2012/12/31"[PDAT]) AND "humans"[MeSH Terms]) AND (English[lang] OR French[lang])

## Haemoperitoneum

("hemoperitoneum"[Mesh] OR "haemoperitoneum"[All Fields] OR "intraperitoneal fluid"[All Fields]) AND "Diagnosis"[Mesh] AND (("1990/01/01"[PDAT] : "2012/12/31"[PDAT]) AND "humans"[MeSH Terms]) AND (English[lang] OR French[lang])

## Complicated ectopic pregnancy

"Pregnancy, Ectopic"[Mesh] AND "Pregnancy, Ectopic/diagnosis"[Mesh] AND (("1990/01/01"[PDAT] : "2012/12/31"[PDAT]) AND "humans"[MeSH Terms]) AND (English[lang] OR French[lang])

## Complicated PID

**1. "Pelvic Inflammatory Disease/diagnosis"[Mesh]**

"Pelvic Inflammatory Disease/diagnosis"[Mesh] AND (("1990/01/01"[PDAT] : "2012/12/31"[PDAT]) AND "humans"[MeSH Terms] AND French[lang]) AND (English[lang] OR French[lang])

**2. MEDLINE**

(tubo[All Fields] AND ovarian[All Fields] AND ("abscess"[MeSH Terms] OR "abscess"[All Fields]) AND ("diagnosis"[Subheading] OR "diagnosis"[All Fields] OR "diagnosis"[MeSH Terms])) AND (("1990/01/01"[PDAT] : "2012/12/31"[PDAT]) AND "humans"[MeSH Terms]) AND (English[lang] OR French[lang])

**Appendix 2**

**Embase search strategy**

EMBASE 1990- December 2012;

## Adnexal torsion

#1

'ovary torsion'/exp/mj/dm_di AND [humans]/lim AND [french]/lim AND [1990-2012]/py

#2

'uterine tube torsion'/exp/mj/dm_di AND [humans]/lim AND [french]/lim AND [1990-2012]/py

#3

#1 OR #2

#4

'ovary torsion'/exp/mj/dm_di AND [humans]/lim AND ([english]/lim OR [french]/lim) AND [1990-2012]/py

#5

'uterine tube torsion'/exp/mj/dm_di AND [humans]/lim AND ([english]/lim OR [french]/lim) AND [1990-2012]/py

#6

#4 OR #5

## Haemoperitoneum

#1

'hemoperitoneum'/exp/mj/dm_di AND [humans]/lim AND [english]/lim AND [1990-2012]/py

#2

'peritoneal fluid'/exp/mj AND 'diagnosis'/exp/mj AND [humans]/lim AND [english]/lim AND [1990-2012]/py

#3

#1 OR #2

#4

'hemoperitoneum'/exp/mj/dm_di AND [humans]/lim AND [french]/lim AND [1990-2012]/py

#5

'peritoneal fluid'/exp/mj AND 'diagnosis'/exp/mj AND [humans]/lim AND [french]/lim AND [1990-2012]/py

#6

#4 OR #5

#7

'hemoperitoneum'/exp/mj/dm_di AND ([english]/lim OR [french]/lim) AND [humans]/lim AND [1990-2012]/py

#9

#7 OR #8

## Complicated ectopic pregnancy

#1

'ectopic pregnancy'/exp/mj/dm_di AND ([english]/lim OR [french]/lim) AND [humans]/lim AND [1990-2012]/py

## Complicated PID

#1

'pelvic inflammatory disease'/exp/mj/dm_di AND [humans]/lim AND ([english]/lim OR [french]/lim) AND [1990-2012]/py

**Appendix 3**

**CENTRAL search strategy**

CENTRAL Issue 2012

## Adnexal torsion

## **#1** - ' **adnexal torsion in title abstract keywords from 1990 to 2012** in **Trials**'

## Haemoperitoneum

## **#1** - **hemoperitoneum and diagnosis in title abstract keywords** in **Trials**

## Complicated ectopic pregnancy

## **#1** '**ectopic pregnancy and diagnosis in title abstract keywords from 1990 to 2012** in **Trials**'

## Complicated PID

**#1** pelvic inflammatory disease and diagnosis:ti,ab,kw from 1990 to 2012, in Trials

**Appendix 4**

**References for the studies included**

**Included studies**

**Haemoperitoneum**

**Chen 1998**

Chen, P. C., G. K. Sickler, *et al*. (1998). "Sonographic detection of echogenic fluid and correlation with culdocentesis in the evaluation of ectopic pregnancy." AJR Am J Roentgenol **170**(5): 1299-1302.

**Fauconnier 2007**

Fauconnier, A., A. Mabrouk, *et al*. (2007). "Ultrasound assessment of haemoperitoneum in ectopic pregnancy: derivation of a prediction model." World J Emerg Surg **2**: 23.

**Glezerman 1992**

Glezerman, M., F. Press, *et al.* (1992). "Culdocentesis is an obsolete diagnostic tool in suspected ectopic pregnancy." Arch Gynecol Obstet **252**(1): 5-9.

**Popowski 2012**

Popowski, T., C. Huchon, *et al*. (2012). "Hemoperitoneum assessment in ectopic pregnancy." Int J Gynaecol Obstet **116**(2): 97-100.

**Sickler 1998**

Sickler, G. K., P. C. Chen, *et al.* (1998). "Free echogenic pelvic fluid: Correlation with hemoperitoneum." Journal of Ultrasound in Medicine **17**(7): 431-435.

**Adnexal torsion**

**Ben-Ami 2002**

Ben-Ami, M., Y. Perlitz, *et al.* (2002). "The effectiveness of spectral and color Doppler in predicting ovarian torsion: A prospective study." European Journal of Obstetrics Gynecology and Reproductive Biology **104**(1): 64-66.

**Cohen 2001**

**Cohen** SB, Wattiez A, Stockheim D, Seidman DS, Lidor AL, Mashiach S, Goldenberg M. The accuracy of serum interleukin-6 and tumour necrosis factor as markers for ovarian torsion. Hum Reprod. 2001 Oct;16(10):2195-7.

**Daponte 2006**

Daponte, A., S. Pournaras, *et al*. (2006). "Novel serum inflammatory markers in patients with adnexal mass who had surgery for ovarian torsion." Fertil Steril **85**(5): 1469-1472.

**Huchon 2010**

Huchon, C., S. Staraci, *et al*. (2010). "Adnexal torsion: a predictive score for pre-operative diagnosis." Hum Reprod **25**(9): 2276-2280.

**Huchon 2012**

Huchon C, Panel P, Kayem G, Schmitz T, Nguyen T, Fauconnier A. Does this woman have adnexal torsion? Hum Reprod. 2012 Aug;27(8):2359-64

**Kupesic 2010**

Kupesic, S. and B. M. Plavsic (2010). "Adnexal torsion: color Doppler and three-dimensional ultrasound." Abdom Imaging **35**(5): 602-606.

**Lee 1998**

Lee, E. J., H. C. Kwon, *et al.* (1998). "Diagnosis of ovarian torsion with color Doppler sonography: depiction of twisted vascular pedicle." J Ultrasound Med **17**(2): 83-89.

**Lee 2011**

Lee, H., W. Lee, *et al.* (2011). "Clinicopathological characteristics of ovarian tumours according to menarchal status in Korean girls." J Paediatr Child Health **47**(7): 436-440.

**Lee 2009**

Lee, J. H., S. B. Park, *et al*. (2009). "Value of intra-adnexal and extra-adnexal computed tomographic imaging features diagnosing torsion of adnexal tumor." J Comput Assist Tomogr **33**(6): 872-876.

**Linam 2007**

Linam, L. E., R. Darolia, *et al.* (2007). "US findings of adnexal torsion in children and adolescents: Size really does matter." Pediatric Radiology **37**(10): 1013-1019.

**Mashiach 2011**

**Mashiach** R, Melamed N, Gilad N, Ben-Shitrit G, Meizner I. Sonographic diagnosis of ovarian torsion: accuracy and predictive factors. J Ultrasound Med. 2011 Sep;30(9):1205-10.

**Mawaldi 2011**

Mawaldi L., Gupta C., H. Bakhsh, M. Saadeh and M. Abolfotouh, "Validity of ultrasound in patients with acute pelvic pain related to suspected ovarian torsion," *Surgical Science*, Vol. 2 No. 6, 2011, pp. 326-330.varian torsion: accuracy and predictive factors. J Ultrasound Med. 2011 Sep;30(9):1205-10.

**Nizar 2009**

Nizar, K., M. Deutsch, *et al*. (2009). "Doppler studies of the ovarian venous blood flow in the diagnosis of adnexal torsion." J Clin Ultrasound **37**(8): 436-439.

**Shiota 2012**

Shiota M, Kotani Y, Umemoto M, Tobiume T, Hoshiai H. Preoperative differentiation between tumor-related ovarian torsion and rupture of ovarian cyst preoperatively diagnosed as benign: a retrospective study. J Obstet Gynaecol Res;39(1):326-9. 2012 Epub ahead of print

**Complicated PID**

**Eschenbach 1997**

Eschenbach, D. A., P. Wolner-Hanssen, *et al*. (1997). "Acute pelvic inflammatory disease: Associations of clinical and laboratory findings with laparoscopic findings." Obstetrics and Gynecology **89**(2): 184-192. **UVSQ**

**Molander 2001**

Molander, P., J. Sjoberg, *et al*. (2001). "Transvaginal power Doppler findings in laparoscopically proven acute pelvic inflammatory disease." Ultrasound Obstet Gynecol **17**(3): 233-238.

**Papavarnavas 1990**

Papavarnavas, C. P., P. F. Venter, *et al*. (1990). "Acute salpingitis - laparoscopic and microbiological evaluation." South African Medical Journal **77**(8): 403-404.

**Rachinsky 2000**

Rachinsky, I., L. Boguslavsky, *et al*. (2000). "Diagnosis of pyogenic pelvic inflammatory diseases by 99mTc-HMPAO leucocyte scintigraphy." Eur J Nucl Med **27**(12): 1774-1777.

**Tukeva 1999**

Tukeva, T. A., H. J. Aronen, *et al*. (1999). "MR imaging in pelvic inflammatory disease: comparison with laparoscopy and US." Radiology **210**(1): 209-216.

**Uslu 2006**

Uslu, H., E. Varoglu, *et al*. (2006). "99mTc-HMPAO labelled leucocyte scintigraphy in the diagnosis of pelvic inflammatory disease." Nucl Med Commun **27**(2): 179-183.

**Complicated ectopic pregnancy**

**Arslan 2004**

Arslan, S., G. Tuncay, *et al.* (2004). "Serum (beta)-hCG level: Can it discriminate between unruptured and ruptured tubal ectopic pregnancies?" Middle East Fertility Society Journal **9**(3): 215-219.

**Atri 1992**

Atri, M., J. de Stempel, *et al*. (1992). "Accuracy of transvaginal ultrasonography for detection of hematosalpinx in ectopic pregnancy." J Clin Ultrasound **20**(4): 255-261.

**Berlingieri 2007**

Berlingieri, P., G. Bogdanskiene, *et al*. (2007). "Rupture of tubal pregnancy in the Vilnius population." Eur J Obstet Gynecol Reprod Biol **131**(1): 85-88.

**Birkhahn 2002**

Birkhahn, R. H., T. J. Gaeta, *et al*. (2002). "Shock index in the first trimester of pregnancy and its relationship to ruptured ectopic pregnancy." Acad Emerg Med **9**(2): 115-119.

**Birkhahn 2003**

Birkhahn, R. H., T. J. Gaeta, *et al*. (2003). "The ability of traditional vital signs and shock index to identify ruptured ectopic pregnancy." Am J Obstet Gynecol **189**(5): 1293-1296.

**Col-Madendag 2010**

Col-Madendag, I., Y. Madendag, *et al*. (2010). "Can sonographic endometrial pattern be an early indicator for tubal ectopic pregnancy and related tubal rupture?" Arch Gynecol Obstet **281**(2): 189-194.

**Develioglu 2002**

Develioglu, O. H., C. Askalli, *et al*. (2002). "Evaluation of serum creatine kinase in ectopic pregnancy with reference to tubal status and histopathology." BJOG **109**(2): 121-128.

**Frates 1994**

Frates, M. C., D. L. Brown, *et al*. (1994). "Tubal rupture in patients with ectopic pregnancy: diagnosis with transvaginal US." Radiology **191**(3): 769-772.

**Goksedef 2011**

Goksedef, B. P., S. Kef, *et al*. (2011). "Risk factors for rupture in tubal ectopic pregnancy: definition of the clinical findings." Eur J Obstet Gynecol Reprod Biol **154**(1): 96-99.

**Hirata 1991**

Hirata, A. J., D. E. Soper, *et al.* (1991). "Ectopic pregnancy in an urban teaching hospital: can tubal rupture be predicted?" South Med J **84**(12): 1467-1469.

**Huchon 2012**

Huchon, C., P. Panel, *et al*. (2012). "Is a standardized questionnaire useful for tubal rupture screening in patients with ectopic pregnancy?" Acad Emerg Med **19**(1): 24-30.

**Job-Spira 1999**

Job-Spira, N., H. Fernandez, *et al*. (1999). "Ruptured tubal ectopic pregnancy: Risk factors and reproductive outcome: Results of a population-based study in France." American Journal of Obstetrics and Gynecology **180**(4): 938-944.

**Malatyalioglu 2006**

Malatyalioglu, E., S. Ozer, *et al.* (2006). "CA-125 levels in ruptured and unruptured tubal ectopic pregnancies." J Obstet Gynaecol Res **32**(4): 422-427.

**Mol 1999**

Mol, B. W. J., P. J. Hajenius, *et al*. (1999). "Can noninvasive diagnostic tools predict tubal rupture or active bleeding in patients with tubal pregnancy?" Fertility and Sterility **71**(1): 167-173.

**Qazi 2010**

Qazi, Q., Z. Akhtar, *et al*. (2010). "Clinical presetations and complications associated with tubal rupture in patients with tubal ectopic pregnancy." JPMI - Journal of Postgraduate Medical Institute **24**(4): 312-317.

**Sadek 1995**

Sadek, A. L. and H. A. Schiotz (1995). "Transvaginal sonography in the management of ectopic pregnancy." Acta Obstet Gynecol Scand **74**(4): 293-296.

**Sadovsky 1991**

Sadovsky, Y., J. Pineda, *et al.* (1991). "Serum CA-125 levels in women with ectopic and intrauterine pregnancies." J Reprod Med **36**(12): 875-878.

**Sindos 2009**

Sindos M, Togia A, Sergentanis TN, Kabagiannis A, Malamas F, Farfaras A, Sergentanis IN, Bassiotou V, Antoniou S. Ruptured ectopic pregnancy: risk factors for a life-threatening condition. Arch Gynecol Obstet. 2009 May;279(5):621-3

**Singh 1992**

Singh, K. B., C. A. Poole, *et al.* (1992). "Characteristics of indigent women with ruptured and unruptured tubal pregnancies." J Reprod Med **37**(8): 745-748.

**van Mello 2012**

van Mello, N. M., C. S. Zietse, *et al*. (2012). "Severe maternal morbidity in ectopic pregnancy is not associated with maternal factors but may be associated with quality of care." Fertil Steril **97**(3): 623-629.

**Appendix 5**

**Flow Chart: Complicated Ectopic Pregnancy**

Records identified through database searching

EMBASE = 2352

Medline = 2558

Central Cochrane = 27

(*N*= 4937)

Additional records identified through other sources
(*n* = 28)

8)

Records after duplicates removed
(*n* = 3819)

**Identification**

Records identified through all sources
(*n* = 4965)

Duplicates removed

(*N*= 1146)

**Screening**

Records excluded on the basis of title and abstract
(*n* = 3656)

**Eligibility**

**Full-text articles assessed for eligibility
(*n* = 163)**

Full-text articles excluded

(*n* = 143)

- No R-EP

**Studies included in qualitative synthesis
(*n* = 20)**

**Included**

**Appendix 6**

**Flow Chart: Complicated Pelvic Inflammatory Disease**

Records identified through database searching

EMBASE = 767

Medline = 930

Central Cochrane = 32

(*N*= 1729)

Additional records identified through other sources
(*n* = 16)

8)

Records after duplicates removed
(*n* = 1613)

**Identification**

Records identified through all sources
(*n* = 1745)

Duplicates removed

(*N*= 132)

**Screening**

Records excluded on the basis of title and abstract
(*n* = 1496)

**Eligibility**

**Full-text articles assessed for eligibility
(*n* = 117)**

Not C-PID= 111

**Included**

**Studies included in qualitative synthesis
(*n* = 6)**

**Appendix 7**

**Flow Chart: Adnexal Torsion**

Records identified through database searching

EMBASE = 116

Medline = 343

Central Cochrane = 1

(*N*= 460)

Additional records identified through other sources
(*n* = 9)

8)

Records after duplicates removed
(*n* = 450)

**Identification**

Records identified through all sources
(*n* = 469)

Duplicates removed

(*N*= 19)

**Screening**

Records excluded on the basis of title and abstract:
(*n* = 408)

**Eligibility**

**Full-text articles assessed for eligibility
(*n* = 42)**

Full-text articles excluded,
(*n* = 28)

**Included**

**Studies included in qualitative synthesis
(*n* = 14)**

**Appendix 8**

**Flow Chart: Haemoperitoneum**

Records identified through database searching

EMBASE 350

Medline 812

Central Cochrane 2

(*N*= 1162)

Additional records identified through other sources
(*n* = 4)

8)

Records after duplicates removed
(*n* = 1055)

**Identification**

Records identified through all sources
(*n* = 1166)

Duplicates removed

(*N*= 111)

**Screening**

Records excluded on the basis of title and abstract:
(*n* = 1035)

**Eligibility**

**Full-text articles assessed for eligibility
(*n* = 20)**

Full-text articles excluded, with reasons
(*n* = 15)

- Not studies of haemoperitoneum diagnosis

**Included**

**Studies included in qualitative synthesis
(*n* = 5)**

**Appendix 9**

**Ultrasound signs**

| **Ultrasound sign/ Condition** | **Study** | **Women (*N*; (D+/D-)^1^** |
| --- | --- | --- |
| **C-PID^2^** | ***N*=4** | ***N*=90 (41/49)** |
| Wall thickness > 5 mm (TVUS^3^) | Molander (2001) | 34 (14/20) |
| Cogwheel sign (TVUS) | Molander (2001) | 34 (14/20) |
| “Beads on a string” (TVUS) | Molander (2001) | 34 (14/20) |
| Incomplete septa (TVUS) | Molander (2001) | 34 (14/20) |
| Cul-de-sac fluid (TVUS) | Molander (2001) | 34 (14/20) |
| Breakdown of adnexal anatomy (TVUS) | Molander (2001) | 34 (14/20) |
| Hyperaemia (Doppler) (TVUS) | Molander (2001) | 34 (14/20) |
| Not specified (TVUS) | Rachinsky (2000) | 16 (8/8) |
| Not specified (TVUS) | Tukeva (1999) | 30 (16/14) |
| Not specified (TVUS) | Uslu (2006) | 10 (3/7) |
| **Haemoperitoneum** | ***N*=4** | ***N*=535 (363/172)** |
| Echogenic fluid (TVUS) | Chen (1998) | 46 (37/9) |
| Echogenic fluid (TVUS) | Fauconnier (2007) | 89 (48/41) |
| Clots (TVUS) | Fauconnier (2007) | 89 (48/41) |
| Fluid around the ovary (TVUS) | Fauconnier (2007) | 89 (48/41) |
| Fluid in the vesico-uterine pouch (TVUS) | Fauconnier (2007) | 89 (48/41) |
| Fluid above the uterine fundus or around the ovary >500 ml (TVUS) | Popowski (2012) | 215 (48/167) |
| Fluid in Morison pouch >500 ml (TAUS^4^) | Popowski (2012) | 215 (48/167) |
| Fluid above the uterine fundus or around the ovary >100 ml (TVUS) | Popowski (2012) | 215 (108/107) |
| Fluid in Morison pouch >100 ml (TAUS) | Popowski (2012) | 215 (108/107) |
| Echogenic fluid (TVUS) | Sickler (1998) | 185 (122/63) |
| **C-EP^5^** | ***N*=6** | ***N*=1303 (251/1052)** |
| Haematosalpinx | Atri (1992 | 18 (6/12) |
| Endometrial pattern: Heterogeneous | Col-Madendag (2010) | 99 (30/69) |
| Endometrial pattern: Homogeneous | Col-Madendag (2010) | 99 (30/69) |
| Endometrial pattern: Trilaminar | Col-Madendag (2010) | 99 (30/69) |
| Intracavity fluid | Col-Madendag (2010) | 99 (30/69) |
| Complex mass | Frates (1994) | 132 (43/89) |
| Tubal ring | Frates (1994) | 132 (43/89) |
| Tubal ring without embryo or yolk sac | Frates (1994) | 132 (43/89) |
| Tubal ring with yolk sac only | Frates (1994) | 132 (43/89) |
| Tubal ring with embryo+ fetal heartbeat | Frates (1994) | 132 (43/89) |
| Tubal ring with embryo without fetal heartbeat | Frates (1994) | 132 (43/89) |
| No mass | Frates (1994) | 132 (43/89) |
| Intraperitoneal fluid (small-large) | Frates (1994) | 132 (43/89) |
| Cul-de-sac fluid (TVUS) | Hirata (1991) | 245 (102/143) |
| Fetal cardiac activity | Mol (1999) | 252 (65/187) |
| Ectopic gestational sac | Mol (1999) | 284 (65/219) |
| Ectopic mass | Mol (1999) | 284 (65/219) |
| Ectopic mass < 24 mm | Mol (1999) | 284 (65/219) |
| Fluid in the pouch of Douglas | Mol (1999) | 284 (65/219) |
| Free pelvic fluid | Sadek (1995) | 525 (5/520^6^) |
| Free pelvic fluid | Sadek (1995) | 53 (5/48^7^) |
| **AT^8^** | ***N*=9** | ***N*=572 (242/330)** |
| Absence of blood flow (Doppler) (TVUS) | Ben-Ami 2002 | 65 (15/50) |
| Absence of blood flow (Doppler) (TVUS) | Kupesic 2010 | 36 (24/12) |
| Twisted vascular pedicle (Doppler) (TVUS) | Lee E 1998 | 47 (32/15) |
| Absence of blood flow (Doppler) (TVUS) | Lee E 1998 | 28 (12/16) |
| Adnexal volume > 20 ml (TA-US) | Linam 2007 | 54 (28/26) |
| Adnexal volume > 75 ml (TA-US) | Linam 2007 | 54 (28/26) |
| Adnexal ratio > 15 (TA-US) | Linam 2007 | 26 (20/6) |
| Absent arterial flow (Doppler) (TA-US) | Linam 2007 | 43 (24/19) |
| Absent or decreased venous flow (Doppler) (TA-US) | Linam 2007 | 43 (24/19) |
| Signs of tissue oedema (Doppler) (TVUS) | Nizar 2009 | 193 (29/164) |
| Absence of intra-ovarian vascularity (Doppler) (TVUS) | Nizar 2009 | 193 (29/164) |
| Absence of ovarian arterial flow (Doppler) (TVUS) | Nizar 2009 | 193 (29/164) |
| Absence of ovarian venous flow (Doppler) (TVUS) | Nizar 2009 | 193 (29/164) |
| Cyst (TVUS+ TA-US) | Mazouni 2005 | 52 (13/39) |
| Hyperechogenicity (TVUS+ TA-US) | Mazouni 2005 | 52 (13/39) |
| Effusion from the Douglas pouch (TVUS+ TA-US) | Mazouni 2005 | 52 (13/39) |
| TVUS | Mawaldi 2011 | 62 (54/8) |
| Ovarian oedema | Mashiach 2011 | 63 (47/16) |
| Abnormal ovarian blood flow | Mashiach 2011 | 63 (47/16) |
| Relative enlargement of ipsilateral ovary | Mashiach 2011 | 63 (47/16) |
| Free fluid around ovary or in Douglas pouch | Mashiach 2011 | 63 (47/16) |
| Ovarian cyst | Mashiach 2011 | 63 (47/16) |
| Abnormal ovary location | Mashiach 2011 | 63 (47/16) |

**1. Disease +/ Disease -; 2. Complicated pelvic inflammatory disease; 3. Transvaginal ultrasound; 4. Trans-abdominal ultrasound; 5. Complicated ectopic pregnancy; 6. The D- group includes** includes intrauterine pregnancies+ non complicated ectopic pregnancies **7. The D- group includes** only non complicated ectopic pregnancies; 8. **Adnexal Torsion;**

**Appendix 10**

**Signs on clinical examination**

| **Clinical examination/ condition** | **Study** | **Women (*N*; (D+/D-)^1^)** |
| --- | --- | --- |
| **C-PID^2^** | ***N*=2** | ***N*=103 (64/39)** |
| Adnexal mass | Eschenbach 1997 | 66 (8/58) |
| Abdominal tenderness | Eschenbach 1997 | 80 (49/31) |
| Severe adnexal tenderness >3 | Eschenbach 1997 | 81 (50/31) |
| Adnexal mass | Eschenbach 1997 | 65 (41/24) |
| Oral temperature > 38°C | Eschenbach 1997 | 81 (50/31) |
| Gainesville clinical stage > grade III | Papavarnavas 1990 | 22 (14/8) |
| **Haemoperitoneum** | ***N*=3** | ***N*=636 (472/154)** |
| Systolic blood pressure < 100 mmHg | Fauconnier 2007 | 89 (48/41) |
| Bimanual examination | Glezerman 1992 | 332 (316/16) |
| Pelvic pain > 4 on NRS | Popowski 2012 | 215 (108/107) |
| Abdominal guarding or rebound tenderness | Popowski 2013 | 215 (48/167) |
| **C-EP^3^** | ***N*=6** | ***N*=1048 (340/708)** |
| Systolic blood pressure < 100 mmHg | Birkham 2002 | 280 (24/256) |
| Heart rate > 100 beats/min | Birkham 2002 | 280 (24/256) |
| Shock index > 0.7 | Birkham 2002 | 280 (24/256) |
| Shock index > 0.85 | Birkham 2002 | 280 (24/256) |
| Heart rate > 100 beats/min | Birkham 2003 | 52 (25/27) |
| Systolic blood pressure < 100 mmHg | Birkham 2003 | 52 (25/27) |
| Shock index > 0.7 | Birkham 2003 | 52 (25/27) |
| Abdominal tenderness | Hirata 1991 | 245 (102/143) |
| Adnexal tenderness | Hirata 1991 | 245 (102/143) |
| Adnexal mass | Hirata 1991 | 245 (102/143) |
| Rebound tenderness | Mol 1999 | 237 (65/172) |
| Vaginal examination - pain | Mol 1999 | 148 (65/83) |
| Vaginal examination - mass | Mol 1999 | 148 (65/83) |
| Shock | Qazi 2010 | 50 (44/6) |
| Abdominal tenderness | Qazi 2010 | 50 (44/6) |
| Adnexal mass | Qazi 2010 | 50 (44/6) |
| Vaginal examination - irregular mass in the pouch of Douglas | Qazi 2010 | 50 (44/6) |
| Adnexal tenderness | Singh 1992 | 184 (145/39) |
| Adnexal mass | Singh 1992 | 184 (145/39) |
| Enlarged uterus > 10 weeks | Singh 1992 | 184 (145/39) |
| **AT^4^** | ***N*=2** | ***N*=164 (88/76)** |
| Adnexal mass | Lee 2011 | 66 (11/55) |
| Abdominal distension | Lee 2011 | 66 (11/55) |
| Acute abdomen | Shiota 2012 | 98 (77/21) |

**1. Disease +/ Disease -; 2. Complicated pelvic inflammatory disease; 3. Complicated ectopic pregnancy; 4. Adnexal Torsion;**

**Appendix 11**

Laboratory tests

| **Biological tests/ condition** | **Study** | **Women (*N*; (D+/D-)^1^)** |
| --- | --- | --- |
| **C-PID^2^** | ***N*=1** | ***N*=76 (47/29)** |
| Leukocytes (> 10,500/µl | Eschenbach 1997 | 76 (47/29) |
| Erythrocyte sedimentation rate (>50) | Eschenbach 1997 | 72 (44/28) |
| C-reactive protein concentration (>1) | Eschenbach 1997 | 46 (24/22) |
| **Haemoperitoneum** | ***N*=2** | ***N*=304 (156/148)** |
| Hb concentration <10 g/dl | Fauconnier 2007 | 89 (48/41) |
| Serum B-hCG concentration < 5000 IU/l | Fauconnier 2007 | 89 (48/41) |
| Hb concentration <10 g/dl | Popowski 2012 | 215 (48/167) |
| Hb concentration <10 g/dl | Popowski 2012 | 215 (108/107) |
| **C-EP^3^** | ***N*=6** | ***N*=696 (258/438)** |
| Creatine kinase > 120 IU/l | Develioglu 2002 | 32 (17/15) |
| B-hCG concentration > 5000 IU/ml | Goksedef 2011 | 232 (88/144) |
| B-hCG concentration < 1500 IU/ml | Goksedef 2011 | 232 (88/144) |
| CA 125 > 21 IU/ml | Malatyalioglu 2006 | 62 (27/35) |
| CA 125 > 34 IU/ml | Malatyalioglu 2006 | 62 (27/65) |
| Serum B-hCG concentration < 1000 IU/l | Mol 1999 | 276 (65/211) |
| Serum B-hCG concentration < 2000 IU/l | Mol 1999 | 276 (65/211) |
| Serum B-hCG concentration < 3500 IU/l | Mol 1999 | 276 (65/211) |
| Serum B-hCG concentration > 3500 IU/l | Mol 1999 | 276 (65/211) |
| Serum Hb concentration 6<7 mmol/l | Mol 1999 | 252 (65/187) |
| Serum Hb concentration < 6 mmol/l | Mol 1999 | 252 (65/187) |
| Hb concentration <10 g/dl | Qazi 2010 | 50 (44/6) |
| Urine test for pregnancy | Qazi 2010 | 40 (34/6) |
| B-hCG concentration > 2000 mIU/l | Qazi 2010 | 6 (5/1) |
| CA 125 > 18 IU/ml | Sadovsky 1991 | 27 (17/10^4^) |
| CA 125 > 18 IU/ml | Sadovsky 1991 | 44 (17/27^5^) |
| **AT^6^** | ***N*=3** | ***N*= 117(34/83)** |
| Serum IL-6 concentration > 10.2 pg/ml | Daponte 2006 | 45 (13/32) |
| Leukocytes > 11000/mm^3^ | Mazouni 2005 | 52 (13/39) |
| Serum IL-6 concentration > 11.3 pg/ml | Cohen 2001 | 20 (8/12) |
| TNF-alfa concentration > 8.1 pg/ml | Cohen 2001 | 20 (8/12) |

**1. Disease +/ Disease -; 2. Complicated pelvic inflammatory disease; 3. Complicated ectopic pregnancy; 4. The D- group includes** includes only non complicated ectopic pregnancies **5. The D- group includes** intrauterine pregnancies+ non complicated ectopic pregnancies; 6. **Adnexal Torsion;**

**Appendix 12**

**Other signs on imaging**

| **Test signs/ condition** | **Study** | **Women (*N*; (D+/D-)^1^** |
| --- | --- | --- |
| **C-PID^2^** | ***N*=3** | ***N*=75 (29/36)** |
| Scintigraphy | Rachinsky 2000 | 20 (8/12) |
| Magnetic raisonance imagery | Tukeva 1999 | 30 (16/14) |
| Scintigraphy | Uslu 2006 | 15 (5/10) |
| **Adnexal torsion** | ***N*=1** |  |
| Abnormal uterine tube thickening (CT^3^) | Lee 2009 | 308 (38/270) |
| Eccentric wall thickening (CT) | Lee 2009 | 282 (28/254) |
| Eccentric septal thickening (CT) | Lee 2009 | 229 (20/209) |
| Decreased/poor contrast enhancement (CT) | Lee 2009 | 308 (38/270) |
| Uterine deviation to the twisted side (CT) | Lee 2009 | 308 (38/270) |
| Negative continuity with gonadal vein (CT) | Lee 2009 | 308 (38/270) |
| Peritumoral infiltration (CT) | Lee 2009 | 308 (38/270) |
| Ascites (CT) | Lee 2009 | 308 (38/270) |

**1. Disease +/ Disease -; 2. Complicated pelvic inflammatory disease; 3. Computed tomography**

PRISMA Checklist

| **Section/topic** | **#** | **Checklist item** | **Reported on page #** |
| --- | --- | --- | --- |
| **TITLE** | | |  |
| Title | 1 | Identify the report as a systematic review, meta-analysis, or both. | 1 |
| **ABSTRACT** | | |  |
| Structured summary | 2 | Provide a structured summary including, as applicable: background; objectives; data sources; study eligibility criteria, participants, and interventions; study appraisal and synthesis methods; results; limitations; conclusions and implications of key findings; systematic review registration number. | 2,3 |
| **INTRODUCTION** | | |  |
| Rationale | 3 | Describe the rationale for the review in the context of what is already known. | 4 |
| Objectives | 4 | Provide an explicit statement of questions being addressed with reference to participants, interventions, comparisons, outcomes, and study design (PICOS). | 5 |
| **METHODS** | | |  |
| Protocol and registration | 5 | Indicate if a review protocol exists, if and where it can be accessed (e.g., Web address), and, if available, provide registration information including registration number. | 6 |
| Eligibility criteria | 6 | Specify study characteristics (e.g., PICOS, length of follow-up) and report characteristics (e.g., years considered, language, publication status) used as criteria for eligibility, giving rationale. | 5, 6 |
| Information sources | 7 | Describe all information sources (e.g., databases with dates of coverage, contact with study authors to identify additional studies) in the search and date last searched. | 6, 7 |
| Search | 8 | Present full electronic search strategy for at least one database, including any limits used, such that it could be repeated. | 6, 7 |
| Study selection | 9 | State the process for selecting studies (i.e., screening, eligibility, included in systematic review, and, if applicable, included in the meta-analysis). | 7 |
| Data collection process | 10 | Describe method of data extraction from reports (e.g., piloted forms, independently, in duplicate) and any processes for obtaining and confirming data from investigators. | 7 |
| Data items | 11 | List and define all variables for which data were sought (e.g., PICOS, funding sources) and any assumptions and simplifications made. | 7 |
| Risk of bias in individual studies | 12 | Describe methods used for assessing risk of bias of individual studies (including specification of whether this was done at the study or outcome level), and how this information is to be used in any data synthesis. | 7 |
| Summary measures | 13 | State the principal summary measures (e.g., risk ratio, difference in means). | 8,9,10,11 |
| Synthesis of results | 14 | Describe the methods of handling data and combining results of studies, if done, including measures of consistency (e.g., I^2^) for each meta-analysis. | NA |
| Risk of bias across studies | 15 | Specify any assessment of risk of bias that may affect the cumulative evidence (e.g., publication bias, selective reporting within studies). | NA |
| Additional analyses | 16 | Describe methods of additional analyses (e.g., sensitivity or subgroup analyses, meta-regression), if done, indicating which were pre-specified. | NA |
| **RESULTS** | | |  |
| Study selection | 17 | Give numbers of studies screened, assessed for eligibility, and included in the review, with reasons for exclusions at each stage, ideally with a flow diagram. | 8 |
| Study characteristics | 18 | For each study, present characteristics for which data were extracted (e.g., study size, PICOS, follow-up period) and provide the citations. | Supporting 18-23 |
| Risk of bias within studies | 19 | Present data on risk of bias of each study and, if available, any outcome level assessment (see item 12). | 9 |
| Results of individual studies | 20 | For all outcomes considered (benefits or harms), present, for each study: (a) simple summary data for each intervention group (b) effect estimates and confidence intervals, ideally with a forest plot. | NA |
| Synthesis of results | 21 | Present results of each meta-analysis done, including confidence intervals and measures of consistency. | NA |
| Risk of bias across studies | 22 | Present results of any assessment of risk of bias across studies (see Item 15). | 8,9 |
| Additional analysis | 23 | Give results of additional analyses, if done (e.g., sensitivity or subgroup analyses, meta-regression [see Item 16]). | NA |
| **DISCUSSION** | | |  |
| Summary of evidence | 24 | Summarize the main findings including the strength of evidence for each main outcome; consider their relevance to key groups (e.g., healthcare providers, users, and policy makers). | 11, 12 |
| Limitations | 25 | Discuss limitations at study and outcome level (e.g., risk of bias), and at review-level (e.g., incomplete retrieval of identified research, reporting bias). | 12,13 |
| Conclusions | 26 | Provide a general interpretation of the results in the context of other evidence, and implications for future research. | 14,15,16,17,18 |
| **FUNDING** | | |  |
| Funding | 27 | Describe sources of funding for the systematic review and other support (e.g., supply of data); role of funders for the systematic review. | 18 |
